# Expression and Function of Bcl-2 Proteins in Melanoma

**DOI:** 10.2174/138920208785699571

**Published:** 2008-09

**Authors:** Jürgen Eberle, Amir M Hossini

**Affiliations:** Charité - Universitätsmedizin Berlin, Department of Dermatology and Allergy, Skin Cancer Center Charité, Berlin, Germany

**Keywords:** Melanoma, apoptosis, Bcl-2 proteins.

## Abstract

Bcl-2 proteins are critical regulators of mitochondrial membrane permeability and the proapoptotic mitochondrial pathway. The family encloses pro- and antiapoptotic factors encoded by over 15 genes, which frequently give rise to alternative splice products. Antiapoptotic, proapoptotic multidomain, and proapoptotic BH3-only proteins are characterized by the presence of at least one of four Bcl-2 homology domains (BH 1-4). Their expression and activities are controlled by survival pathways as MAP kinases and protein kinase B/Akt, which are in touch with a number of transcription factors. In melanoma, the mitochondrial apoptosis pathways and Bcl-2 proteins appear of particular importance for apoptosis resistance, which has been addressed in clinical trials applying antisense-Bcl-2. Overexpression or induction of proapoptotic Bcl-2 proteins as well as the use of small molecule mimetics for the proapoptotic BH3 domain are further promising strategies.

## INTRODUCTION

Cancer is a predominant cause of death worldwide, and its importance is even further increasing due to the generally extending life expectancy. Currently one in four deaths in the United States and in Europe attributes to cancer, and in an age range between 45–65, the proportion further increases up to 50% [1, 2]. This attributes to the fact that the treatment options for disseminated cancer are largely insufficient.

A notable example is malignant melanoma of the skin, which is characterized by an unbroken high mortality with median survival rates of only 8 months in its disseminated stage. Patients can be cured only by early and complete surgical excision of the primary tumor, whereas in disseminated disease systemic therapies as various chemotherapies, biotherapies or vaccination protocols so far were not able to improve the dismal prognosis [3-5].

Though different mechanisms may contribute to chemoresistance, defective apoptosis signaling may be the predominant cause in cancer [6, 7]. This corresponds to the fact that cancer therapies in general aim at the elimination of tumor cells through apoptotic processes. Bcl-2 proteins, which will be the topic of this review, are major players in the regulation of apoptosis, and melanoma will be used as an example for discussing therapeutic strategies for targeting Bcl-2 proteins.

## INTRINSIC PATHWAYS TO APOPTOSIS

In contrast to necrosis as a kind of accidental cell death, apoptosis is an active and physiological process indispensable for normal tissue homeostasis, which acts as a counterbalance for cell proliferation [8]. Beyond this, it provides fundamental safeguard mechanisms in protection from cancer by eliminating altered and potentially harmful cells.

**Fig. (1). Bcl-2 proteins in the control of the mitochondrial apoptosis pathway.**
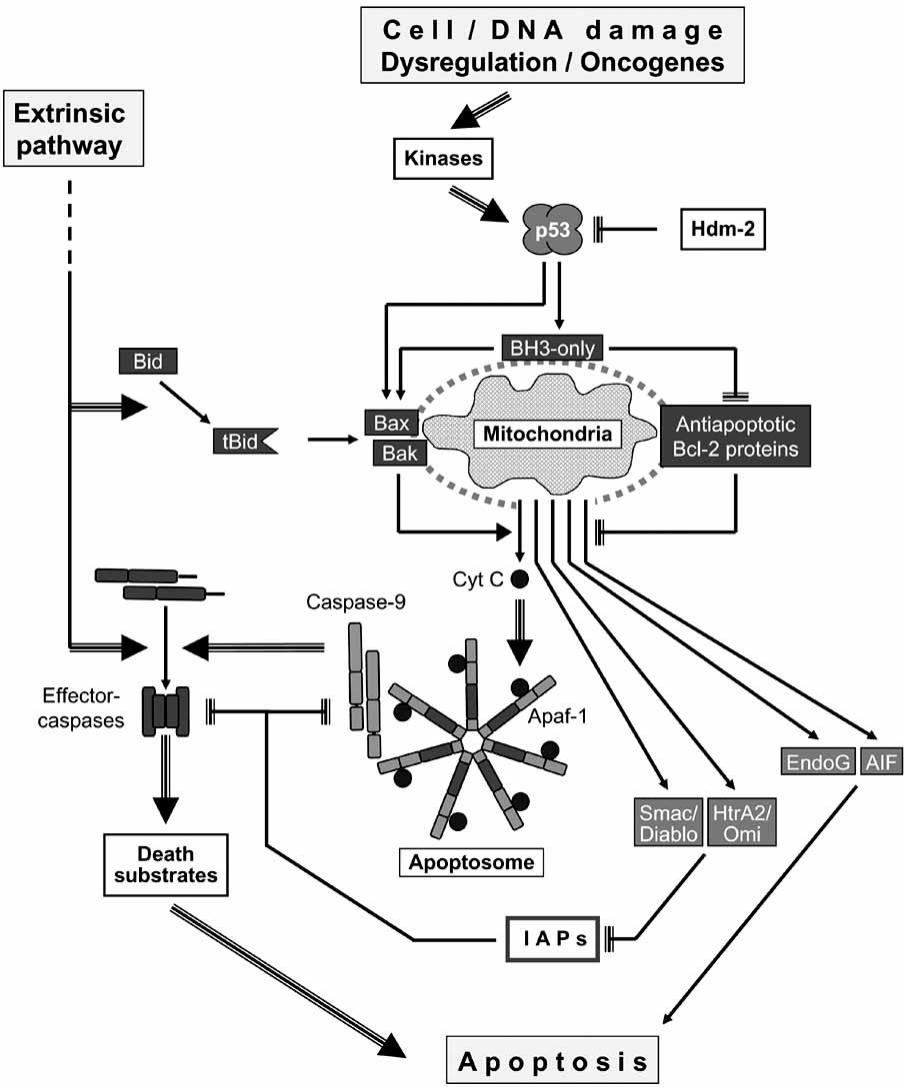
Abbreviations: Cyt C, cytochrome C; IAPs, inhibitors of apoptosis proteins; tBid, truncated Bid; BH3-only, BH3-only proteins; AIF, apoptosis-inducing factor; EndoG, endonuclease G; Apaf-1, apoptosis-activating factor.

Different types of cellular damage as well as cellular dysregulation such as oncogene activation induce cell-intrinsic proapoptotic pathways. Highly active is the mitochondrial pathway characterized by a release of mitochondrial proapoptotic factors into the cytoplasm [9]. This step is critically controlled by the family of Bcl-2 proteins (Fig. **1**).

As a master regulator in apoptosis, the transcription factor p53 triggers intrinsic pathways. Levels of p53 are kept low in healthy cells due to its short half-life and degradation by the proteasome pathway. Here the ubiquitin ligase Hdm2 exerts an important function [10]. In response to DNA damage, hypoxia, oncogene activation or other intrinsic problems, p53 is stabilized by several possible modification steps such as phosphorylation by ATM (ataxia telangiectasia-mutated), ATR (ATM- and rad3-related) or checkpoint kinases (Chk1, Chk2) [11]. As a transcription factor, p53 drives the expression of various proteins including the cell cycle inhibitor p21, which results in a stop of cell division, thus providing time for DNA repair. In case of irreparable damage however, p53 triggers the transcription of proapoptotic factors as in particular proapoptotic Bcl-2 proteins such as Bax, Noxa, Puma, Bik/Nbk and Bid. Furthermore, cytosolic p53 may also directly interact with Bcl-2 family members to trigger mitochondrial apoptosis [12-14].

The family of pro- and antiapoptotic Bcl-2 proteins critically controls the permeability of organelles as the outer mitochondrial membrane [15]. Once a certain threshold of proapoptotic signals is exceeded, a rapid release of mitochondrial intermembrane factors is induced. Several factors exert characteristic proapoptotic functions when emerging in the cytoplasm, as reported for cytochrome C, endonuclease G, AIF (apoptosis-inducing factor), Smac/DIABLO (second mitochondria-derived activator of caspases/direct IAP-binding protein with low pI) and HtrA2/Omi [16].

Cytochrome C triggers the formation of the apoptosome, a multiprotein complex enclosing each seven copies of the adaptor protein Apaf-1 (apoptosis-activating factor), Cyt C and ATP [17]. It allows the binding and activation of initiator caspase-9, which sets up a subsequent caspase cascade starting with caspase-3. Caspases (Cysteine-dependent aspartate directed poteases) represent hallmarks in apoptosis. They are synthesized as inactive zymogens, and they activate each other through cleavage processes thus forming signal cascades. Proapoptotic caspases are divided up in initiator caspases (2, 8, 9 and 10) and effector caspases (3, 6, and 7) [18, 19].

Effector caspases themselves have hundreds of different cellular protein targets (death substrates), which are inactivated or activated upon cleavage [20]. Thus, CAD (caspase-activated DNase) in healthy cells is hold in check by binding to ICAD (inhibitor of CAD). In course of apoptosis, CAD is released by caspase-mediated cleavage of ICAD resulting in a cleavage of DNA between nucleosomes visible as a characteristic DNA ladder [21]. In parallel, the DNA repair enzyme PARP (poly ADP-ribose polymerase) is inactivated upon caspase cleavage ensuring that energy resources are reserved for apoptosis as well as that DNA repair does not occur simultaneously to DNA fragmentation [22]. The result of such changes is the complete reprogramming of the cell for apoptosis.

The proapoptotic potential of other mitochondrial factors is related to the family of inhibitor of apoptosis proteins (IAPs). Their function in apoptosis is to block caspase-9 and effector caspases, which is achieved by binding to their active sites and/or by ubiquitination and targeting caspase degradation. Both Smac/Diablo and HtrA2/Omi have been described to augment caspase activation by antagonizing IAP proteins [23, 24]. In contrast, the activities of AIF and endonuclease G appear as largely independent from caspase pathways. After translocation to the cytosol and to the nucleus, their proapoptotic contribution depends on an endogeneous DNAse activity (Endo G) or DNA interaction, chromatin condensation and recruitment of downstream nucleases in case of AIF [25].

Thus, the mitochondrial membrane appears as a place for integration of different pro- and antiapoptotic signals, where the decision about life and death is made. The release of proapoptotic proteins appears as the critical step, which depends on the equilibrium between pro- and antiapoptotic Bcl-2 proteins. Proapoptotic Bcl-2 proteins support the mitochondrial permeability while the antiapoptotic proteins try to block it. 

## STRUCTURE OF Bcl-2 PROTEINS AND GENOMIC ORGANIZATION

**Fig. (2). Bcl-2 family proteins.**
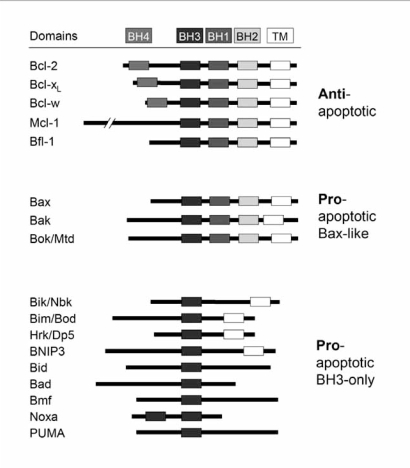
The superfamily of Bcl-2 proteins encloses antiapoptotic factors, proapoptotic Bax-like factors and BH3-only proteins. The presense of up to four Bcl-2 homology domains (BH 1-4) as well as in some proteins the transmembrane domain (TM) is indicated.

Bcl-2 proteins are master regulators of the mitochondrial pathway. More than twenty known proteins of the family (incl. alternative splice products) are categorized according to their function (anti or proapoptotic) and according to the presence of up to four Bcl-2 homology domains (BH1 – BH4). Most antiapoptotic proteins as Bcl-2 or Bcl-x_L_ share all four domains, whereas proapoptotic proteins separate into and multidomain proteins (Bax, Bak and Bok), which share BH 1, 2, 3 BH3-only proteins, which have only BH3(Fig. **2**). In addition there are other proteins, which do not fit in these categories.

As concerning the genomic organization of the more than 15 genes encoding for Bcl-2 proteins, most of them are located on different chromosomes. Clustering is seen only occasionally, i.e. Bax and Puma reside in 19q13, and Bcl-2 and Noxa are in 18q21 (Table **1**).

## MODELS FOR EXPLAINING THE REGULATION OF APOPTOSIS BY Bcl-2 PROTEINS

Despite intensive research since the identification of Bcl-2 in 1984 by the t(14;18) translocation in acute B-cell leukaemia [26], the exact mechanism(s), how Bcl-2 proteins control mitochondrial permeability, remain partly elusive. Different models have been suggested. Accordingly, the multidomain proapoptotic proteins are in healthy cells in an inactive conformation and either cytosolic (Bax) or only loosely attached to mitochondria (Bak). Upon induction of apoptosis, they are activated, undergo conformational changes and integrate deeper into the outer mitochondrial membrane, to either form channels themselves or trigger opening of the permeability transition pore (PTP) [27, 28].

In contrast, several antiapoptotic Bcl-2 proteins are permanent constituents of the mitochondrial membrane, where they protect membrane integrity. They bind to multidomain proteins Bax and Bak and thus block their proapoptotic activity. Supporting the model, Bcl-2 or Bcl-x_L_ have been shown to prevent Bax translocation and activation [29-31], and Bcl-x_L_ and Mcl-1 have been shown to sequester Bak in mitochondria [32].

The BH3-only proteins are regarded as triggers in apoptosis control. They are transactivated or post-translationally activated by protein modifications in response to diverse apoptotic stimuli. Thus, Bid is activated by proteolytic cleavage through caspase-8 or granzyme B, Bad is activated upon dephosphorylation and Bim is released from cytoskeletal structures [33-35]. Upon activation, they bind to the antiapoptotic Bcl-2 proteins thus to neutralize their activity and leading to a freeing of Bax and Bak [36]. BH3 is required for the interaction and binds into a hydrophobic pocket formed by BH1, BH2 and BH3 of antiapoptotic Bcl-2 proteins, thus leading to their neutralization (Adams and Cory, 2007).

Some BH3-only proteins display selective binding to specific antiapoptotic Bcl-2 family members as Bad, which interacts with Bcl-2, Bcl-w and Bcl-x_L_ but not with Mcl-1, or Noxa, which binds to Mcl-1 and Bfl-1 but not Bcl-2 and Bcl-x_L_. Others as Puma and Bim may be less selective and bind to all antiapoptotic Bcl-2 proteins [37]. Thus more than one BH3-only protein may be required for induction of apoptosis, as demonstrated in HeLa cells, where the release of Bak from Bcl-x_L_ and Mcl-1 required activation of Bad and Noxa [38]. Antiapoptotic Bcl-2 family members were shown to sequester BH3-only proteins at the mitochondria. Thus tBid and Bad were found in complex with Bcl-2 and Bcl-x_L_ [39], and Bim was found in complex with Mcl-1, which was disrupted upon induction of apoptosis [40, 41].

Alternative models suggests that BH3-only proteins may also directly interact with Bax and Bak for their activation [42], however fluorescence imaging could not demonstrate their co-localization [43]. Besides their control of apoptosis and besides their localization in mitochondria, Bcl-2 proteins are also located in the endoplasmatic reticulum (ER), where they are involved in diverse cellular processes, like calcium homeostasis, autophagy, the unfolded protein response and ER morphogenesis [44].

## ALTERNATIVE SPLICING

Some members of the Bcl-2 family are regulated by alternative splicing. A prominent example is Bim, which occurres as a short (Bim_S_), a long (Bim_L_) and an extra long protein (Bim_EL_). All three proteins promote apoptosis, but have distinct activities and distinct modes of regulation conferred by their interaction with other proteins [45]. Even further splice products of Bim have been reported with undetermined function. Also for Bax eight splice variants have been reported, which have partly different domain structures, of which 6 have been shown to exert proapoptotic functions [46].

Also several antiapoptotic Bcl-2 proteins are controlled by alternative splicing. Thus for Bcl-2, an alternative splice product of so far unknown function has been reported (Bcl-2β), which lacks the transmembrane domain and preferentially locates to the cytosol [47]. Also Bfl-1 has a short alternative splice variant (Bfl-1_S_), which localizes to the nucleus due to an intrinsic nuclear localization sequence. Also Bfl-1_S_ revealed antiapoptotic activities, as determined in coexpression experiments with Bax [48].

Finally the bcl-x gene is expressed in at least five reported isoforms of different activities. While Bcl-x_L _(long), Bcl-xβ and Bcl-x_ES_ (extra short) are antiapoptotic [49-51], Bcl-x_S_ (short) and Bcl-x_AK_ (alternative killer) exert proapoptotic functions. Most interestingly, Bcl-x_AK_ is the first proapoptotic Bcl-2 protein that lacks the BH3 domain [52], which so far has been regarded as indispensable for the proapoptotic function [36]. Thus even other ways of apoptosis regulation by Bcl-2 proteins have to be considered.

Alternative splicing of Bcl-2 protein mRNAs seems to be regulated by different stimuli, which strongly depended on the cell type investigated. Thus for the example of Bcl-x proteins, glucocorticoids and progestins may selectively trigger bcl-x_L_ mRNA expression and influence the ratio between bcl-x_L_ and bcl-x_S_ [53]. In glioma cells, Bcl-x_L_ levels were increased by TPA, whereas interleukin-6 or GM-CSF reduced the proportion of Bcl-x_L_ in leukemia cells [54].

As a selecting factor, the RNA binding protein Sam68 has been described. Depletion of Sam68 by siRNA caused accumulation of antiapoptotic Bcl-x_L_, whereas Sam68 up-regulation increased the levels of Bcl-x_S_ [55].

## Bcl-2 PROTEINS AND MELANOMA APOPTOSIS RESISTANCE

The mitochrondrial apoptosis pathways appear of particular importance for melanoma and are activated in response to diverse stimuli also including death ligands [56-58]. Antiapoptotic proteins as Bcl-2, Bcl-x_L_ and Mcl-1 are highly expressed [59-61], and a high Bcl-2/Bax ratio correlated with apoptosis resistance [62].

Regulation of the expression of Bcl-2 has been attributed to microphthalmia-associated transcription factor (MITF) [63], whereas Bcl-x_L_ expression may depend on NF-*κ*B activity [64]. Indicative for its critical role, Bcl-2 overexpression reduced basic apoptosis and sensitivity of melanoma cells for proapoptotic stimuli [65, 66]. Despite the well accepted role of Bcl-2 in melanocyte survival, its contribution to chemoresistance of metastasized melanoma remains unclear, because high Bcl-2 expression in primary melanomas did not correlate with a worsening of prognosis [67], and even reduced expression levels were found in metastases, where Bcl-x_L_ and Mcl-1 were upregulated [68].

Also expression of proapoptotic family members was found in melanoma cells as Bax, Bak, Bid, Bad, PUMA and Noxa [69-71], whereas others as Bcl-x_S_ and Bik/Nbk were lacking [72, 73]. Proapoptotic Bcl-2 proteins may also be upregulated in course of chemotherapy. Thus, taurolidine-induced apoptosis in melanomas correlated with enhanced Bax and reduced Bcl-2 expression [74], and bortezomib induced expression of Noxa [75]. Also a prognostic value of proapoptotic Bcl-2 proteins was found, namely down-regulated Bax and Bak in primary melanomas correlated with unfavorable prognosis [76].

## TRANSCRIPTIONAL REGULATION AND SIGNAL CASCADES CONTROLLING Bcl-2 PROTEINS 

Constitutive activation of central survival pathways as mitogen-activated protein kinases (MAPKs), protein kinase B (PKB/Akt) and nuclear factor-kappaB (NF-κ B) characteristically occur in cancer cells, and substantial evidence connects them with chemoresistance [77]. The canonical MAPK pathway is activated downstream of receptor tyrosine kinases (RTK) and results in successive phosphorylation of Raf, MEK (MAP/extracellular signal-regulated kinase) and ERK1/2 (extracellular signal-regulated kinases) [78]. On the other hand, Akt is activated by either RTKs or G-protein-coupled receptors [79]. MAPKs and Akt mediate the phosphorylation of multiple apoptosis regulators, and MAPKs in addition lead to the activation of many different transcription factors. Especially, both Akt and ERK may result in activation of the NF-κ B pathway, which triggers the induction of antiapoptotic factors such as IAP proteins and Bcl-x_L_ [80].

Different cell death signals as well as growth factor deprivation are linked with a regulation of several Bcl-2 proteins. Thus, Bax, Noxa, Puma, Bik/Nbk and Bid are upregulated by p53 [81-83]. Bim can be upregulated by FoxO-3A, CEBPα (CCAAT-enhancer binding protein-α) or CHOP (CEBP homologous protein) [84, 85]. In contrast, transcriptional up-regulation of antiapoptotic Bcl-2 proteins often depends on survival signals, as Bcl-x_L_ is up-regulated by the JAK-STAT pathway [86].

Several Bcl-2 proteins are in addition regulated by protein modification. Thus Mcl-1 is characterized by an only short half-life due to its degradation through the proteasome pathway, which can be slowed down by phosphorylation through ERK [87]. In contrast, Bim is inactivated by ERK [88], while Bax and Bad are phosphorylated and inactivated by either ERK or Akt [89, 90]. Hereby, either phosphorylation of Bad, by MAPKs (serine 112) or Akt (serine 136), is sufficient for its inhibition [91]. In contrast, phosphorylation of Bax by Glycogen synthase kinase-3beta (GSK-3β) promotes its activation [92]. Also Bak is activated in response to stress-activated protein kinases as MEKK1 and JNK1, but it seems not to be phosphorylated itself. Intermediate regulators as possibly BH3-only proteins may be employed [93].

Melanoma is characterized by constitutive ERK1/2 expression [94, 95], which may result from activating mutations in *B-Raf* (60%) and *N-Ras* (30%) [96, 97]. Thus inactivation of Bad and Bim through MAPK phosphorylation as well as downregulation of PUMA and upregulation of Mcl-1 by MAPKs can be assumed as contributing to melanoma cell survival and chemoresistance [98-100].

The transcription factors activated by MAPKs in melanocytic cells enclose MITF, which particularly accounts for high Bcl-2 expression [101], as well as factors of the Ets or CREB/ATF families, which may be upregulated by MAPKs and induce Bcl-2 and/or Bcl-x_L_ [102-104]. Other pro-survival activities in melanoma cells have been reported for ATF-1, ATF-2 and CREB [105, 106].

Several antiapoptotic activities of PKB/Akt have been identified in melanoma cells, such as Akt-mediated phosphorylation of Bad [107] and activation of the NF-κB pathway through an Akt-mediated pathway [108]. 

## THERAPEUTIC STRATEGIES BASED ON Bcl-2 PROTEINS AND CONCLUSIONS

Because of the critical role of the mitochondrial pathway in melanoma, approaches targeting anti- and proapoptotic Bcl-2 proteins are of particular interest. This may be achieved by targeting survival pathways, due to their control over the Bcl-2 protein expression. Thus, applying MAPK inhibitors induced basic apoptosis and sensitized for pro-apoptotic strategies, which correlated to activation of Bad and of Bax *in vitro* as well as in mouse models [109-111].

Proteasome inhibitors were applied to induce apoptosis *via* inhibition of NF-κB. In addition, recent evidence suggested a critical contribution of up-regulation of NOXA which appeared early after proteasome inhibition and correlated with apoptosis [112, 113]. As a contrary effect however, also antiapoptotic Bcl-2 proteins may be upregulated as Mcl-1, which is degraded by the proteasome pathway. Thus, pro- and antiapoptotic Bcl-2 proteins upregulated by proteasome inhibitors appear in balance, and better therapeutic effects may be obtained with suitable combinations, as recently shown for Mcl-1 siRNA [114, 115].

Approaches directly targeting Bcl-2 proteins in melanoma appear of particular interest. Thus, Bcl-2 antisense oligonucleotide strategies were established. Both *in vitro* and in mouse models, melanoma cells were sensitized for the chemotherapeutic dacarbazine [116]. Also, phase I/II clinical trials showed positive results [117], and a large phase III trial (dacarbazine + Bcl-2 antisense), completed in 2003, showed improvements of the clinical response. Significant improvement of the overall survival was found, however, only in a subgroup of patients with low serum LDH [118].

Complicating an antisense strategy, Bcl-2 expression may also be reduced in metastatic melanoma [119], and other antiapoptotic Bcl-2 proteins such as Mcl-1 or Bcl-x_L_ may substitute for Bcl-2 [120]. Also, antisense strategies have been developed for these proteins, which similarly enhanced chemosensitivity *in vitro* and in mouse models [121, 122]. Due to high expression of several antiapoptotic Bcl-2 proteins in melanoma, a simultaneous targeting may be necessary [119, 123], which may however be difficult to realize in the clinic. Other approaches used oligonucleotides directed against specific splice sites as the 5'-splice site of Bcl-x_L_, which resulted in reduced ratio of Bcl-x_L_ to Bcl-x_S_ in breast cancer cells [124].

As pro- and antiapoptotic Bcl-2 proteins are in balance to control the mitochondrial pathway, the overexpression of proapoptotic Bcl-2 proteins appears as an alternative strategy for the targeting of antiapoptotic factors. The efficacy of such strategies has been demonstrated in several *in vitro* studies, where apoptosis was efficiently induced in melanoma cells and chemosensitivity was increased by the exogeneous overexpression of Bcl-x_S_, Bik/NBK, Bax, Bcl-x_AK_ or Noxa [125-128].

Related with such strategies, new developments try to mimic the BH3 domain of proapoptotic Bcl-2 proteins, which is supposed to bear the main proapoptotic potential [129]. These BH3 mimetics are peptides or small molecules structurally related to different BH3 domains, and depending on their structure, they reveal distinct specificities for blocking different antiapoptotic Bcl-2 proteins [130].

Gossypol is a naturally occurring BH3 mimetic isolated from cotton seeds, which binds Bcl-x_L_ and Bcl-2. It triggered apoptosis even in Bcl-2- or Bcl-x_L_-overexpressing cells or such cells that were deficient for both Bax and Bak, which are otherwise resistant to chemotherapy [131, 132]. Also in melanoma cell lines, Gossypol efficiently induced cell death [133]. Another example that induced apoptosis in melanoma, lymphoma and pancreatic carcinoma cells is a Bim-related BH3 domain linked to the HIV TAT protein for better membrane transduction (TAT-Bim) [134].

Much work in different tumors has been done with the small molecule BH3-mimetic ABT-737, which was identified by systematic screening [135]. It inhibits Bcl-2, Bcl-x_L_ and Bcl-w but appears as inactive against Mcl-1 and A1/Bfl-1. Several studies demonstrated a sensitization of tumor cells for chemotherapy [136-139] or, as shown in melanoma cells, it enhanced the proapoptotic effects of co-cultured T-cells [140].

In particular, resistance to MAPK inhibition based on high levels of antiapoptotic Bcl-2 proteins was overcome in melanoma cells by another small molecule BH3 mimetic (TW-37) developed by computer modeling for binding to Mcl-1, Bcl-x_L_ and Bcl-2 [141]. The computer-based design of BH3 mimetics may even allow a further improvement of their efficacy, particularly the simultaneous targeting of all antiapoptotic Bcl-2 proteins is aimed [142].

In summary, dysregulation of Bcl-2 proteins appears of critical importance for melanoma cell survival and drug resistance, and among the fast increasing number of new targeted therapies, those, which affect Bcl-2 proteins, may especially apply for melanoma. These are in particular: i) blocking of survival pathways to control Bcl-2 protein expression, ii) antisense strategies against antiapoptotic Bcl-2 proteins, iii) gene therapy approaches based on overexpression of proapoptotic Bcl-2 proteins and iv) further development of BH3 mimetics. Finally, as a large body of preclinical and clinical evidence demonstrates, highly aggressive tumors as melanoma may not be defeated by monotherapies, rather combinations of several signaling effectors should be envisioned. The challenge of the future will be to identify suitable combinations which will also prove efficient in the clinic, and strategies targeting Bcl-2 proteins will play a dominant role.

## Figures and Tables

**Fig. (3). Survival pathways involved in regulation of Bcl-2 proteins.**
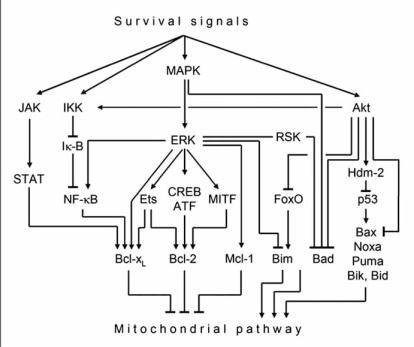
Four survival pathways (MAPK, PKB-Akt, IKK, JAK/Stat) are indicated which are linked to a number of transcription factors. Some transcription factors are listed here (NF-κB, Ets1/2, CREB/ATF family, MITF and FoxO, p53, STAT), which control the expression of Bcl-2 proteins.

**Table 1.** Characteristics of Bcl-2 Proteins

Proteins	Chr. Location 1	Splicing 2	Activation 3
***Antiapoptotic***			
Bcl-2	18q21.3	2	Ph
Bcl-w	14q11.2 - q12	n.r.	
Mcl-1	1q21	2	
Bcl-x_L_	20pter-p12.1	5	Deph / Ph
Bfl-1	15q24.3	2	
***Proapoptotic***			
Bax	19q13.3 - q13.4	8	Deph / Ph / p53
Bak	6p21.3	n.r.	Con
Bid	22q11.2	4	Cl / p53
Bad	11cen-q12.3	n.r.	Deph
Nbk/Bik	22q13.2-q13.3	n.r.	Ph / p53
Hrk	12q24.21	n.r.	
Bim	2q12-q13	10	Ph
Bmf	15q14	n.r.	Ph
Puma	19q13.3 - q13.4	2	p53
Noxa	18q21.32	n.r.	p53

1 Chromosomal location of bcl-2 genes.

2 Reported numbers of alternative splice products. For several splice variants, there is no function so far identified, as for several splice variants of Bim and two splice variants of Bax. For several proteins the number of splice products has not been reported (n.r.).

3 Mechanisms for activation of Bcl-2 proteins: Ph, phosphorylation; Deph, dephosphorylation; Cl, cleavage; Con, conformational change; p53, transactivation by p53.
